# Financial Crisis: A New Measure for Risk of Pension Fund Portfolios

**DOI:** 10.1371/journal.pone.0129471

**Published:** 2015-06-18

**Authors:** Marinella Cadoni, Roberta Melis, Alessandro Trudda

**Affiliations:** 1 Department of Political Science, Communication, Engineering and Information Technologies, University of Sassari, Sassari, Italy; 2 Department of Economics, Universitas Mercatorum, Rome, Italy; 3 Department of Economics and Business, University of Sassari, Sassari, Italy; Universidad Veracruzana, MEXICO

## Abstract

It has been argued that pension funds should have limitations on their asset allocation, based on the risk profile of the different financial instruments available on the financial markets. This issue proves to be highly relevant at times of market crisis, when a regulation establishing limits to risk taking for pension funds could prevent defaults. In this paper we present a framework for evaluating the risk level of a single financial instrument or a portfolio. By assuming that the log asset returns can be described by a multifractional Brownian motion, we evaluate the risk using the time dependent Hurst parameter *H*(*t*) which models volatility. To provide a measure of the risk, we model the Hurst parameter with a random variable with mixture of beta distribution. We prove the efficacy of the methodology by implementing it on different risk level financial instruments and portfolios.

## Introduction

Pension funds control large capitals and represent the biggest institutional investors in many countries. Despite their social security function, their performance is usually measured in terms of returns rather than risk. The 2008 financial crisis saw the default of some of the biggest pension funds worldwide, highlighting the inadequacy of current performance measures. The case of the fund CalPERS (California Public Employees’ Retirement System) is emblematic: by focusing on high rate-of-investment-returns whilst overlooking risk levels it suffered combined losses of 67 billion in 2008 and 2009, amounting to more than a third of its capitalization being forced to impose an increase in contributions to cover the losses.

Often, rules on pension funds investments are derived from laws that regulate speculative investment companies. As a consequence, qualitative rules are used to classify the risks of individual financial products according to their typology: liquidity, bonds, stocks, derivatives, commodities etc. Otherwise, limits may be related to geographical areas, where usually non-OECD countries are considered highly risky. Following the financial crisis it has been shown how this classification can be misleading (the case of the Greek bonds is exemplary).

This calls for quantitative limits for investment in pension funds and measures that analyze quantitatively the degree of risk of pension portfolios.

Otranto and Trudda (2007) [1] sustain the need for a classification of risk for pension funds and propose a cluster analysis based on the GARCH volatility of the return rate. Bianchi and Trudda (2008)[2] analyze the investment risk in pension funds, developing a technique for rebalancing pension funds portfolios in function of their pointwise level of risk. The global asset log return is modeled by a multifractional Brownian motion. The aim of our paper is to model the investment risk and our framework is based on that of Bianchi and Trudda (see [2]). We define the risk as the *roughness* of the returns of the price series and we model the log returns of a price series by a Multifractional Brownian Motion with random exponent (On the same topic see also [3]).

Our main contribution consists in interpreting the Hölder exponent as a random variable *H* and in deriving the salient characteristics of its distribution to provide a measure of the risk. The roughness of the process is then represented by the Hölder exponent, which can assume values in the interval [0, 1]. When 0 < *H*(*t*) < 1/2 the roughness level is high, indicating a “turbulence”of the returns of the portfolio. Conversely, when 1/2 < *H* < 1 the return process is “quieter”.

In the application, seven investment portfolios are simulated to show how the levels of risk obtained can be very different.

## Methods

To describe the log price dynamics we use a multifractional process with random exponent (MPRE). Before describing it, let us first introduce the fractional Brownian motion (fBm) presented by Kolmogorov in 1940 [4] and defined in the seminal paper by Mandelbrot and Van Ness in 1968 [5], whose multifractional Brownian motion is a generalization (On this topic see also [6]).

### Fractional and Multifractional Brownian Motion

The fBm is characterized by a slowly decaying autocorrelation function depending on the *Hurst* exponent *H* ∈ (0,1]. Following the definition that can be found in [7], the process has moving average representation

$$\begin{array}{ccc} B_{H}\left(t\right) & = & C{\left\{\pi K\left(2H\right)\right\}}^{1/2}\int_{R}f_{t}\left(s\right)dB\left(s\right) \end{array}$$ (1)

with

$$\begin{array}{ccc} f_{t}\left(s\right) & = & \frac{1}{\Gamma \;\left(H+\frac{1}{2}\right)}\left\{{\left|t-s\right|}^{H-\frac{1}{2}}1_{]-\infty ,t]}\left(s\right)-{\left|s\right|}^{H-\frac{1}{2}}1_{]-\infty ,0]}\left(s\right)\right\} \end{array}$$

where *B*(⋅) stands for the ordinary Brownian motion, *C* is a positive constant and *K* is the function defined on [0, 2] as $K(\alpha )=\Gamma (\alpha +1)\frac{\text{sin}\frac{\alpha \pi }{2}}{\pi }$. The process is self-similar of parameter *H* and has stationary increments. Its covariance function reads as

$$\begin{array}{c} E\left(B_{H}\left(t\right)B_{H}\left(s\right)\right)=\frac{c^{2}}{2}\left({\left|t\right|}^{2H}+{\left|s\right|}^{2H}-{\left|t-s\right|}^{2H}\right) \end{array}$$ (2)

(A process *X*(*t*), *t* ∈ *T* is defined *self-similar* with parameter *H* if for any *α* > 0 $\left\{X(\alpha t)\}\overset{d}{=}\{\alpha^{H}X(t)\right\}$, where the equality holds for the finite-dimensional distributions of the process (see [8] and [9])).

The *multifractional Brownian motion* (mBm, see [10], [11], [12]) is a generalization of the fBm obtained by allowing *H* to vary over time and has the following representation

$$\begin{array}{ccc} M_{H(t)}\left(t\right) & = & C{\left\{\pi K\left(2H\left(t\right)\right)\right\}}^{1/2}\int_{R}f_{t}\left(s\right)dB\left(s\right) \end{array}$$ (3)

with

$$\begin{array}{ccc} f_{t}\left(s\right) & = & \frac{1}{\Gamma \;\left(H\left(t\right)+\frac{1}{2}\right)}\left\{{\left|t-s\right|}^{H\left(t\right)-\frac{1}{2}}1_{]-\infty ,t]}\left(s\right)-{\left|s\right|}^{H\left(t\right)-\frac{1}{2}}1_{]-\infty ,0]}\left(s\right)\right\} \end{array}$$

where *H* : [0, ∞) → (0, 1] is required to be a Hölder function of order 0 < *η* ≤ 1 to ensure the continuity of the motion.

Since *H*(*t*) is the punctual Hölder exponent of the mBm at point *t*, the process is locally asymptotically self-similar with index *H*(*t*) (see, e.g., Benassi et al.[13])) in the sense that, denoted by *Z*(*t*, *au*) ≔ *M*_*H*(*t*+*au*)_(*t* + *au*) − *M*_*H*(*t*)_(*t*) the increment process of the mBm at time *t* and lag *au*, it holds

$$\begin{array}{c} \lim_{a\to 0^{+}}a^{-H(t)}Z\left(t,au\right)\overset{d}{=}B_{H(t)}\left(u\right)\text{,}\;u\in R. \end{array}$$ (4)

The above distributional equality indicates that at any point *t* there exists an fBm with parameter *H*(*t*) tangent to the mBm. Furthermore, since *B*_*H*(*t*)_(*u*) ∼ 𝓝(0, *C* ^2^ *u* ^2*H*(*t*)^), the infinitesimal increment of the mBm at time *t*, normalized by *a* ^*H*(*t*)^, normally distributes with mean 0 and variance *C* ^2^ *u* ^2*H*(*t*)^ (*u* ∈ *R* → *a* → 0^+^).

The increments of the mBm are no longer stationary nor self-similar; despite this, the process is extremely versatile since the time dependency of *H* is useful to model phenomena whose punctual regularity is time changing.

In our context, *H*(*t*) can represent the degree of confidence the investors nourish in the past. High values of *H*(*t*) correspond to trends (or low volatility phases), i.e. to periods in which the past information weighs in the investors’ trading decisions; low values of *H*(*t*) are associated to high volatility periods, in which prices display an anti-persistent or mean reverting behaviour because of the quick buy-and-sell activity that is typically induced by uncertainty. Standard financial theory is recovered when $H=\frac{1}{2}$, case in which the mBm reduces to the Brownian motion.

### Our proposed model

By allowing *H* to be a stochastic process or a random variable, the mBm can be further generalized to the Multifractional Process with Random Exponent. Ayache and Taqqu (see [14]) define it following an intuition of Papanicolau and Solna ([15]) proposing to replace the deterministic functional parameter H(t) of the mbm by a stochastic process.

Let: *H*:[0, 1] → [*a*, *b*] ⊂ [0, 1] be a random or stochastic process, *B*_*H*_:[0, 1] × [*a*, *b*] ⊂ (0,1) a Gaussian field and *f*_1_:[0, 1] → [0, 1] × [*a*, *b*], *t* → (*t*, *H*(*t*, *w*)) and $f_{2}:[0,1]\times [a,b]\to \text{R},(t,H)\to B_{H}(t,w)$. Then the process is defined by:

$$\begin{array}{c} Z\left(t,w\right)=f_{2}\left(f_{1}\left(t\right)\right)=B_{H(t,w)}\left(t,w\right) \end{array}$$

Here follow the salient properties of the MPRE that inspired our model:

1. (Ayache and Taqqu [14])—The pointwise Hölder exponent of the MPRE is determined by *H*(*t*, *w*) almost surely. Since the Hölder regularity of the MPRE process is still determined by the Hölder exponent, we can evaluate the roughness of the series by using *H*(*t*, *w*).
2. (Bianchi [16])—*H*(*t*, *w*) can be interpreted as the confidence level investors have in past information. The Hölder exponent summarises the weights that investors nourish in the past and this is conditional to the new information that spreads in the market. High values of *H*(*t*) correspond to trends (or low volatility phases), i.e. to periods in which the past information weighs in the investors’ trading decisions; low values of *H*(*t*) are associated to high volatility periods, in which prices display an anti-persistent or mean reverting behaviour because of the quick buy-and-sell activity that is typically induced by uncertainty. As a consequence, *H* is not symmetrical with respect to its central value 1/2 as it is easier (quicker) to loose confidence than to build it.
3. (Ayache, Taqqu [14])—If *H* is a random variable independent of the Brownian motion then the process is stationary. The stationarity is therefore a necessary condition for *H* to be a random variable independent of the Brownian motion.

From the listed properties it follows that, provided we are dealing with stationary processes, we can model *H* with a random variable and this will give us a measure of the roughness and so the risk of a price series. Given the asymmetry of *H* w.r.t. its central value of 1/2 and its range in the interval [0, 1], we choose a random variable with *beta* distribution. Indeed, the *beta* distribution presents some characteristics that mime very well those of *H*.

Our proposed methodology to evaluate the risk of a price series can be summarized in the following steps:

1. *Establish the stationarity of the series of returns*
  To accomplish this we used the Dickey-Fuller roots of unity test. All series of returns used in the experiments were stationary according to the test.
2. *Define the risk as the roughness of the series of returns*
  A high level of roughness of a return series is the sign of an intense buy and sell activity, the higher the peaks and falls, the higher the uncertainty the operators express on the asset. Conversely, a stable behaviour of the price series, with a low roughness level, means that, over time, the market has deemed the asset to be low risk, resulting in stable prices. Therefore, recalling that a constant value of H = 0.5 corresponds to a classical Brownian motion, when the series of returns has a high level of roughness (corresponding to H values less than 0.5), the asses is perceived as risky and its price is highly volatile. On the other side, a low level of roughness (corresponding to H values greater than 0.5), means that prices are not turbulent, i.e., the market considers the asset to be low risk.
3. *Model the series of returns by a MPRE and the roughness by the Hölder exponent*
  At this stage, we need to estimate the Hölder exponent from real data. To do so, we adopt a family of “moving-window” estimators of *H*(*t*) based on the *k*-th absolute moment of a Gaussian random variable of mean zero and given variance *V*_*H*_ (the variance of the unit lag increment of a mBm) as defined in [16] (see also [10]). Given a series of length *N* and a window of length *δ*, the estimator has the form
  $$\begin{array}{ccc} H_{\delta ,N}^{k}\left(t\right) & = & \frac{\log(2^{k/2}\Gamma (\frac{k+1}{2})V_{H}^{k/2})-\log(\frac{\sqrt{\pi }}{\delta }\sum_{j=t-\delta }^{t-1}{|X_{j+1,N}-X_{j,N}|}^{k})}{k\log(N-1)} \end{array}$$ (5)
  for *j* = *t* − *δ*, …, *t* − 1; *t* = *δ* + 1, …, *N*; *k* ≥ 1.
  The quick rate of convergence $O(\delta^{-\frac{1}{2}}{\left(\text{log}\;N\right)}^{-1})$), the estimator is reliable even for very short *δ*′ *s*. Toilsome computations show that when $H=\frac{1}{2}$ the variance of the estimator reduces to
  $$Var(H_{\delta ,N}^{k}(t))=\frac{\sqrt{\pi }}{\delta k^{2}\;{\text{ln}}^{2}\;\left(N-1\right)\;{\left[\Gamma \;\left(\frac{k+1}{2}\right)\right]}^{2}}\cdot (\Gamma \;\left(\frac{2k+1}{2}\right)-\frac{1}{\sqrt{\pi }}{\left[\Gamma \;\left(\frac{k+1}{2}\right)\right]}^{2})$$ (6)
  and the optimal value of *k* is deduced by minimizing the last relation. (6) reaches a minimum for *k* = 2, so this is the value we set in our experiments.
4. *Model *H* with a random variable with a mixture of beta distributions*
  The *Beta* distribution, being defined in the interval [0, 1] and being dependent on two parameters, has the potential to well approximate the Hölder exponent.
  The probability density function of the *beta* distribution is given by:
  $$\begin{array}{c} y=f\left(x,a,b\right)=\frac{1}{B(a,b)}x^{a-1}{\left(1-x\right)}^{b-1} \end{array}$$
  where $B(a,b)=\frac{\Gamma (a+b)}{\Gamma (a)\Gamma (b)}$ is the *beta* function.
  Its mean and variance are given by:
  $$\begin{array}{c} \mu =\frac{a}{a+b}\;\;\;\;\;\;\;\;\;\;\;\;\;Var=\frac{ab}{\left(a+b+1\right){\left(a+b\right)}^{2}} \end{array}$$
  To better model possible bimodal characters of the Hölder exponent we adopt a linear combination of two *Beta* distributions:
  $$\begin{array}{c} \alpha_{1}f\left(x,a_{1},b_{1}\right)+\alpha_{2}f\left(x,a_{2},b_{2}\right) \end{array}$$
  with *α*_1_ + *α*_2_ = 1.
5. *Use the parameters of the distribution as a risk measure*
  The parameters *a* and *b*, of the *beta* or the mixture of *beta* provide us with the moments of the random variable *H*. In particular, we could use *b* as a direct measure of the risk of the price series: the higher the value of *b* the smaller the risk and viceversa.

## Results and discussion

After establishing how well the mixture of *beta* distribution approximates the Hölder exponent, we apply our model on seven low risk and high risk portfolios.

### Mixture of *beta* goodness of fit

To evaluate the goodness of fit of the mixture of *beta* distribution we derived *H* from 20 daily price series over a time span of 5 years, we fitted the Hurst parameter with a mixture of Beta and performed the Kolmogorov-Smirnov test (K-S test). The K-S test is based on the maximum distance between the empirical distribution function of a sample and the cumulative distribution function of a continuous distribution. Since it evaluates the maximum distance between the two distributions, the test is quite stringent and can detect different shapes particularly in the central body of the distribution. On the other hand, when the test is used as a goodness of fit test, if the parameters of the distribution are obtained from the sample itself, the K-S statistics are not reliable, and a Monte Carlo procedure is necessary to obtain accurate p-values. Since we obtain the parameters of the mixture of distributions by a maximum likelihood fit on the sample data, we calculate the initial K-S statistic between the Hölder exponent sequence and the linear combination of Beta distributions fitted on it, and we use the initial statistic to run a Monte Carlo simulation according to the method by Clauset et al. [17]. The p-value threshold suggested in [17] is 0.1.

The assets used are listed in the first column of Table 1 and include market indexes (Bovespa, Athen Index Compos, Hang Seng Index (HSI), Ibex, Dow Jones, NASDAQ, FTSE), some shares listed in those indexes (Cheung Kong Holdings Ltd, Bank of China, Petroleo Brasileiro, Telecom) a portfolio made of bonds of emerging countries (Arca Bonds Emergenti) and EU bonds: Belgium Kingdom-BGB, Bundesrepub. Deutschlan-DBR, Irish TSY-Irish, Intesa San Paolo SPA-ISPIM, Netherlands Government-Nether, Obrigacoes do Tesouro-PGB, Republic of Austria-RAGB, Telecom Italia FIN SA-TITIM.

**Table 1:** K-S test results for single assets.

**Assets**	***a*_1_**	***b*_1_**	***a*_2_**	***b*_2_**	***w***	**p value**
Cheung Kong Holdings	24.72	21.05	43.87	35.29	0.4994	**0.32**
Bank of China	41.29	34.69	18.58	14.55	0.4760	0.02
Arca Bonds Emergenti	21.17	3.35	6.33	1.31	0.8088	**0.15**
Athen	10.34	13.72	58.33	47.44	0.1196	**0.11**
Petroleo Brasileiro	122.55	60.96	57.16	47.86	0.2023	0.03
Ibex	218.42	138.94	31.17	22.83	0.2755	0.05
Telecom	51.62	50.29	63.17	46.17	0.3418	**0.26**
Bovespa	102.37	155.31	52.32	38.33	0.0275	0.003
HSI	174.15	97.08	34.69	27.81	0.2780	**0.31**
DJI	249.10	93.53	26.56	16.17	0.0957	**0.10**
NASDAQ	343.85	465.29	50.54	33.25	0.0202	0.05
FTSE	172.22	194.77	40.87	26.42	0.1138	**0.23**
BGB	39.40	7.98	70.21	17.76	0.4987	**0.10**
DBR	298.04	89.55	73.87	16.84	0.1654	**0.34**
Irish	32.67	18.66	59.19	15.70	0.2084	**0.53**
ISPIM	54.06	21.33	63.80	13.77	0.274	**0.37**
Nether	81.17	19.41	94.28	15.50	0.755	0.02
PGB	21.56	10.87	153.55	45.90	0.4884	**0.32**
RAGB	27.27	42.55	55.64	12.30	9.12e-10	**0.25**
TITIM	35.74	16.12	141.028	52.40	0.4999	**0.31**

**Fig 1:**
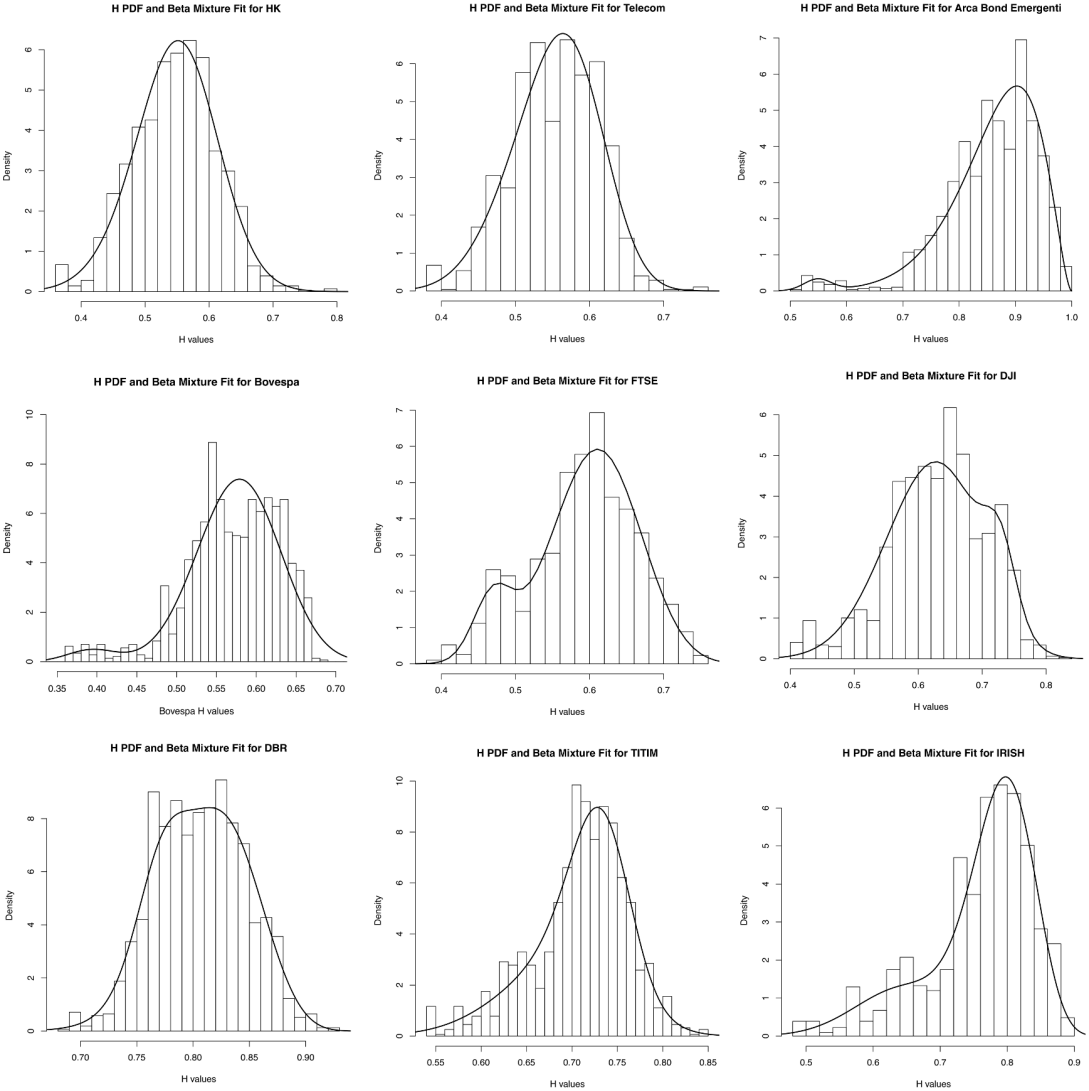
Mixture of beta distribution fit to the Hölder exponent of different types of assets. The Hölder exponents are represented by histograms whose number of bins is determined by the Freedman-Diaconis rule, the probability density functions of the mixtures of *beta* are the black curves.

Fig 1 shows the fitting of the mixture of distribution on two shares, a portfolio of bonds of emerging countries, three indexes and three EU bonds. In the first row of pictures of Fig 1 are shown two shares and a found (Arca Bonds Emergenti), in the second three market indexes and in the third three Eurozone bonds. The images and the K-Stest p-values show that the model fits excellently the *H* values of the assets Cheung Kong Holdings, DBR, IRISH, TITIM, FTSE, fits well Telecom, Arca Bond Emergenti and DJI, and fits poorly the index Bovespa. More precisely, in the case of the Bovespa index, the linear combination of two *beta* accurately models the tail of the distribution of *H* but cannot fit properly the central part, indicating more parameters would be needed to model *H* effectively. From a financial point of view, we can notice how the indexes are modelled by *beta* distributions which have their global maximum for *H* values in the range (5,6), indicating a high probability that the asset will encounter a high turbulence phase, while for the EU bonds and the Arca Emergenti (composed by emerging countries bonds), the global maximum of the density of the distribution is assumed for *H* values in the range (7,9), so it is likely that *H* will assume those values, which means that a low risk is associated to the asset. In the table, columns 2 to 5 are the parameters of the mixture of *beta*: *wf*(*x*, *a*_1_, *b*_1_) + (1 − *w*)*f*(*x*, *a*_2_, *b*_2_). In the last column, the p-values after the Monte Carlo simulation are recorded. P-values greater than 0.1 are highlighted in bold, which amount to 14 out of 20 assets (70% of the analyzed cases). The p-value is greater than 0.02 in five other cases and only for the index Bovespa is smaller. The mixture of beta fits well the majority of the Hölder exponent sequences of the assets analyzed.

### Application to pension founds portfolios

To evaluate the performance of the method we simulated seven portfolios. The financial assets contained in the portfolios are daily time series from January 2006 to December 2011, which includes the 2007–2008 market crisis. Three portfolios are made entirely of shares and indexes and so are labelled as high risk portfolios (HRP1,HRP2, HRP3) in Table 2, four portfolios are made of bonds of various countries and are labelled as low risk portfolios (LRP1, LRP2, LRP3, LRP4). The assets of each portfolio are specified in the second column of Table 2. If no percentage is specified, the weights of the assets are equally distributed.

**Table 2:** Portfolios composition.

**Portfolio**	**Assets in portfolio**
HRP1	Athen, Bovespa, Ibex, HSI, Cheung Kong Holdings Ltd, Bank of China, Brasil Petroleo, Telecom
HRP2	CPFE, HSI, Natu3
HRP3	Ibex, Telecom, Bovespa, HSI
LRP1	90% Arca Bond Emergenti, 10% Cheung Kong Holdings Ltd
LRP2	Irish, ISPIM, TITIM
LRP3	BGB, DBR, RAGB
LRP4	BGB, DBR, Irish, ISPIM, Nether, PGB, RAGB, TITIM.

**Fig 2:**
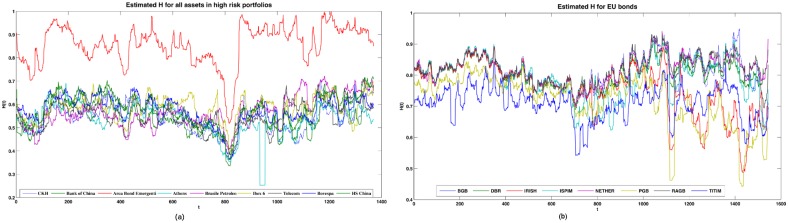
H of the assets. (a) *H* of the high risk assets: the indexes, shares and the Arca Bond Emergenti portfolio. (b) *H* of the low risk assets: the EU area bonds.

For each asset in the portfolios we estimated *H* with a moving window of 20 days lag. In Fig 2(a) we can see the values of *H* for the high risk market indexes and shares and for the Arca Bond Emergenti portfolio made of emerging countries bonds, in Fig 2(b) *H* of the low risk assets, the EU area bonds. The EU bonds prove to be less risky than the shares, as expected, whereas the portfolio of bonds from emerging countries (Arca Bonds Emergenti), despite being generally perceived as high risk, is the one with the highest values of *H*.

**Fig 3:**
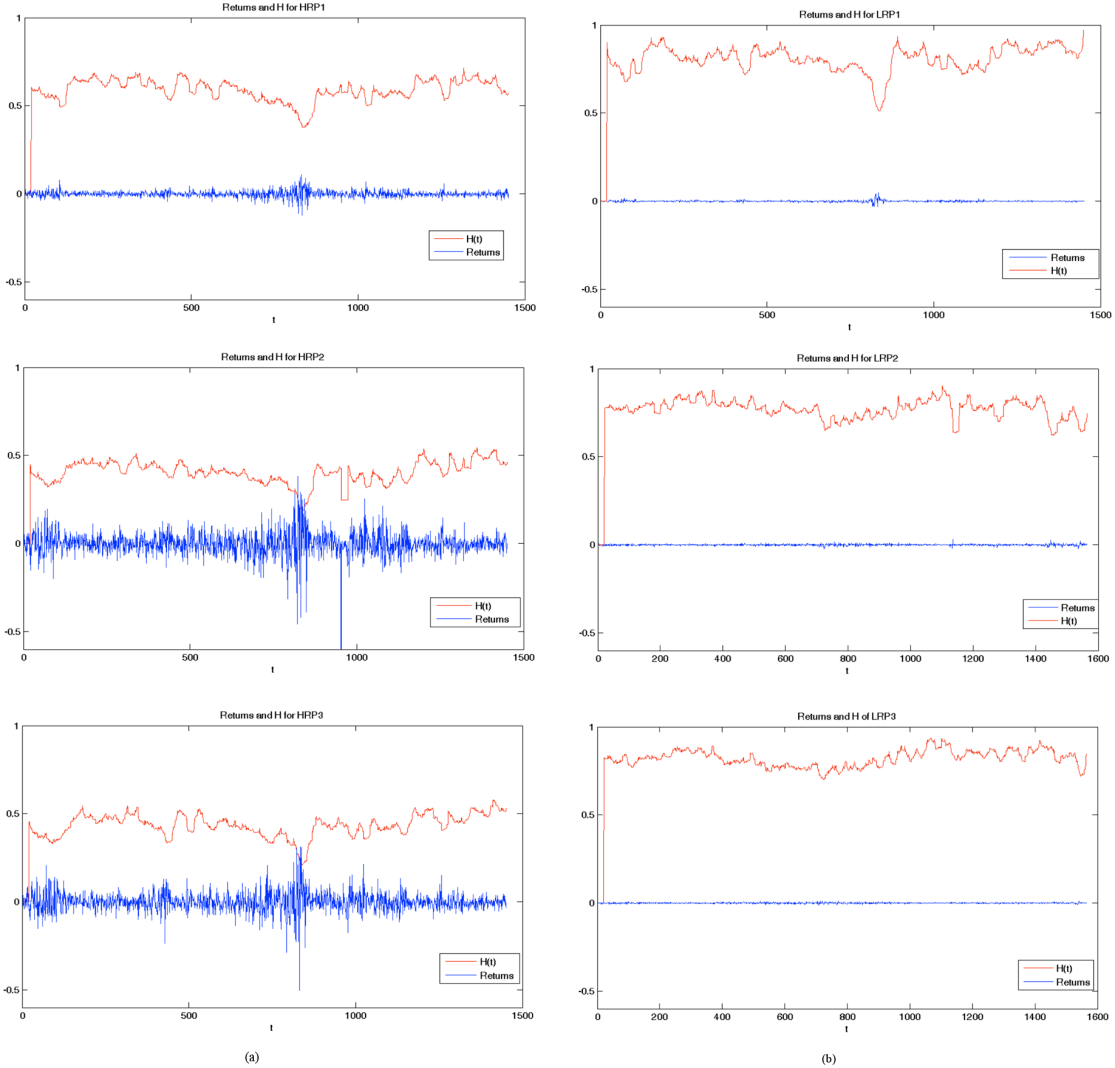
*H* and returns of the portfolios. Estimated *H* and returns for three high risk portfolios (column (a)) and three low risk portfolios (column (b)). The lag for the estimation of H was set to 20 days for all portfolios.

To estimate *H* for the portfolios, we summed up the return series of which the portfolios are composed and then calculated the value of *H*. Fig 3, column (a), shows the high risk portfolios are composed by assets with numerous turbulence phases and so the values of *H* are definitely lower than those of the low risk portfolios (column (b)) whose return series are much more stable.

**Fig 4:**
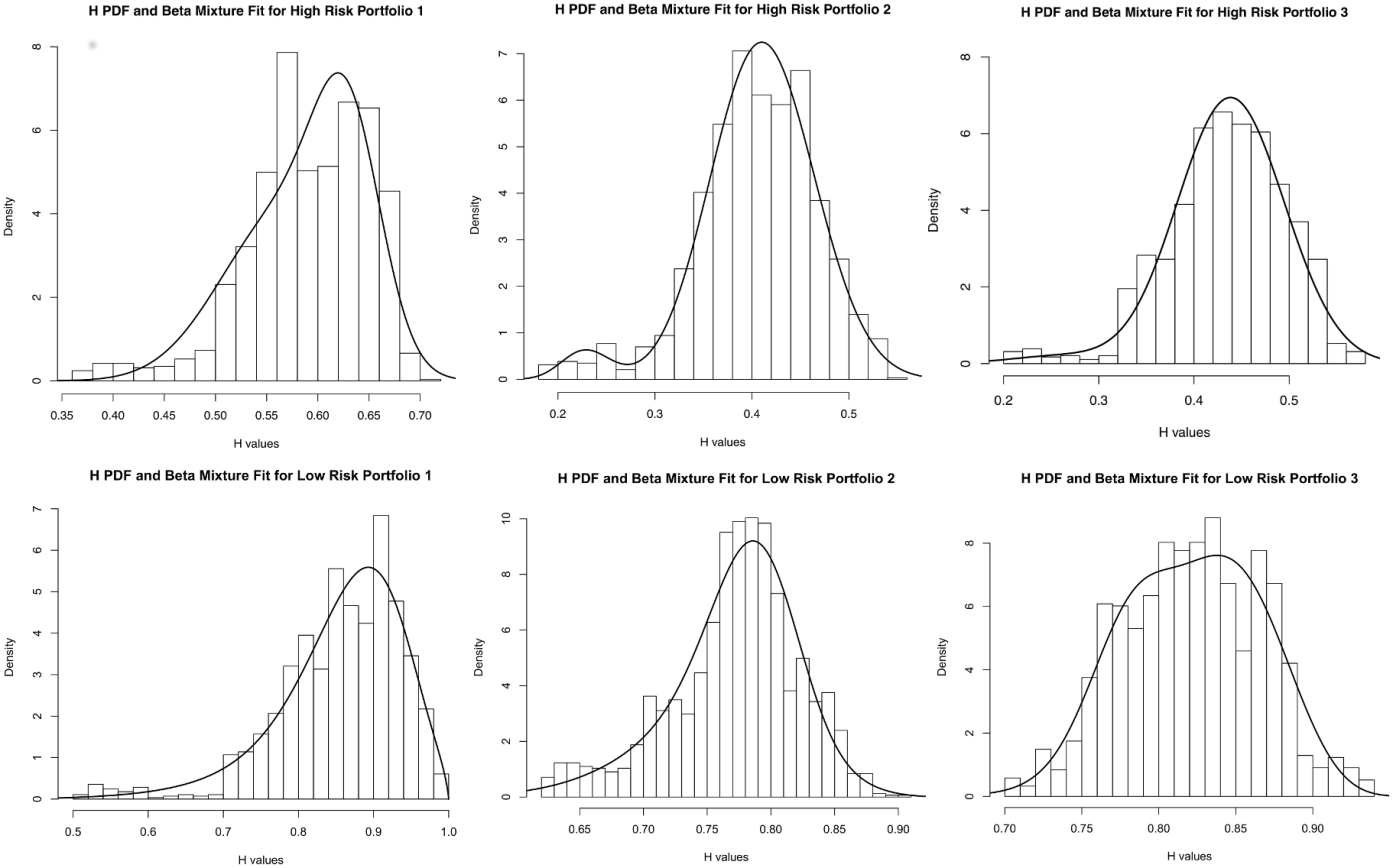
Mixture of beta distribution fit to the Hölder exponent of different portfolios. First row: mixture of *beta* fitting of *H* of high risk portfolios (HRP1, HRP2, HRP3). Second row: mixture of *beta* fitting of *H* of low risk portfolios (LRP1, LRP2, LRP3).

We then fitted the *beta* distribution to find the parameters that give a quantitative measure of the risk. As it can be seen from Table 3, the K-S test is positive for six out of seven portfolios. The distribution fittings are shown in Fig 4.

**Table 3:** KS test results for all portfolios.

**Portfolio**	***a*_1_**	***b*_1_**	***a*_2_**	***b*_2_**	***w***	**p value**
HRP1	135.50	80.43	38.63	30.00	0.4115	0.005
HRP2	65.53	218.98	35.86	51.13	0.0392	0.17
HRP3	14.57	36.81	35.3911	45.03	0.0358	0.242
LRP1	26.74	4.29	9.999	1.77	0.4962	0.123
LRP2	35.56	11.34	123.58	33.91	0.4999	0.2791
LRP3	208.18	59.90	62.44	12.55	0.2004	0.305
LRP4	43.79	7.22	77.51	19.49	0.1345	0.344

**Fig 5:**
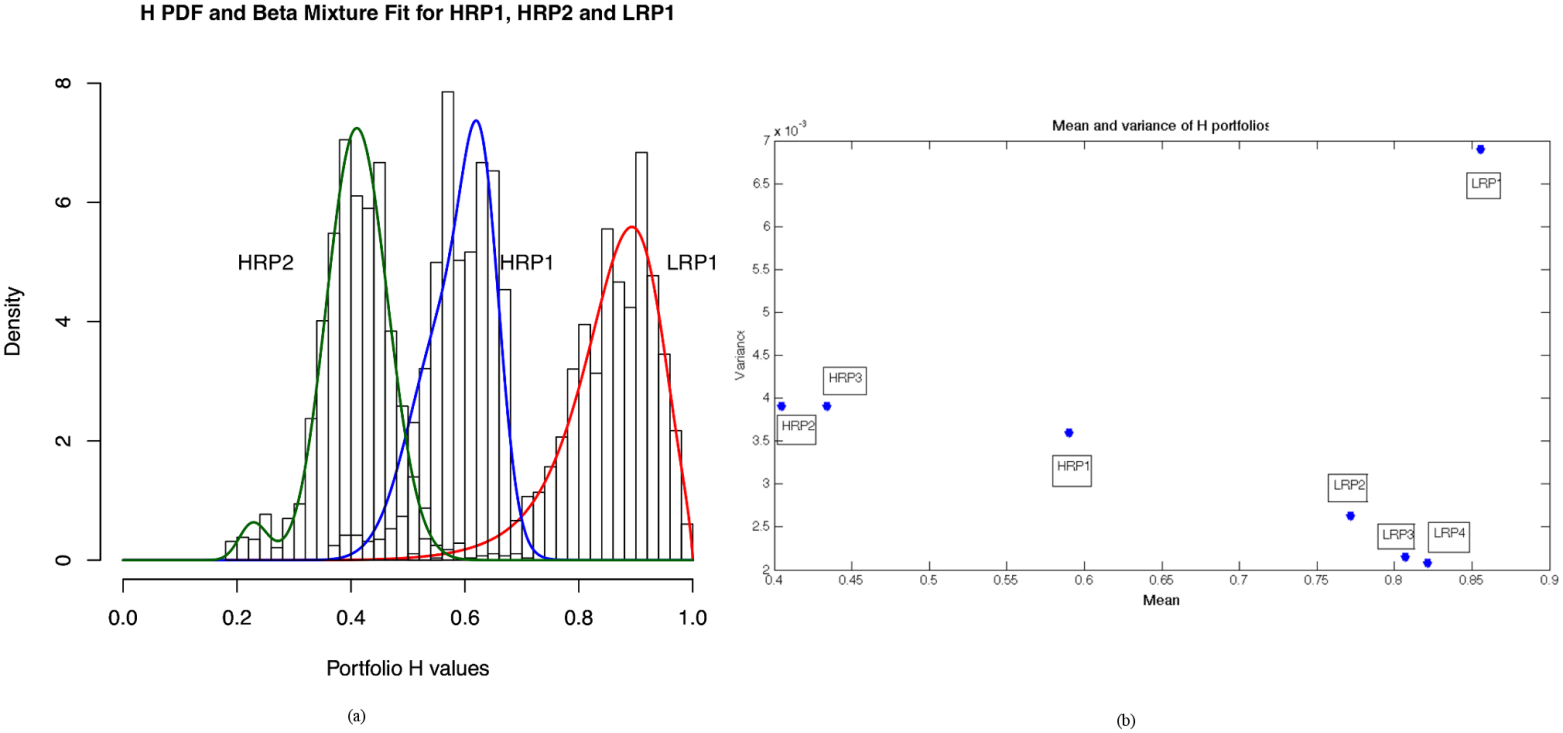
(a) Mixture of beta distribution fit to the Hölder exponent of High Risk Portfolio 2, High Risk Portfolio 1, and Low Risk Portfolio 1. (b) Mean and variance of the *beta mixture* distribution for the seven portfolios.

Fig 5a) compares the distributions of *H* of three portfolios, two composed of high risk assets (distributions on the left and center), and the other of low risk assets (distribution on the right). In Fig 5b) the plot of the mean and variance relative to the random variable *H* of the seven portfolios confirms that the low risk portfolios have *H* values near one. Moreover, it is interesting we notice that LPR1, which is composed mainly of non OECD bonds, has a higher variance than all the others porfolios. This is due to the fact that the assets in it, though having low risk profiles, tend to go through transitory turbulences associated to the macroeconomics trends of emerging countries and to currency exchanges fluctuations. In contrast, the high risk portfolios show a low level of variability in the values taken by *H*, underlining a sort of “time invariance” in the volatility levels attributed to them.

## Conclusions

In 2008 a market crisis caused the failure of major pension funds worldwide. Several analysis show that the funds tend to increase the portfolios risk in order to obtain higher values of the expected global asset return while many authors emphasize that pension funds have to maintain a prudent profile because the social function (in particular for the first pillar) prevails over the speculative function. In general, financial laws use mutual fund regulations to determine the limits of investments in risky financial instruments. Moreover, regulations are often qualitative and do not use quantitative methods.

We proposed a method to quantitatively assess the risk of pension funds investments, which consists of modeling the log return series with a MPRE and the risk with the Hölder exponent *H* of the process. High values of *H*, near one, indicate a low roughness level and so a low level of volatility associated to the portfolio; conversely, low values of *H*, near zero, are associated to series with a high level of roughness, which in terms of markets translates in a feverish buy and sell activity (high volatility). To quantify the risk we model *H* with a random variable with *beta* distribution, which depends on two parameters that can vary to fit very well the asymmetry of *H*, at the same time providing us with different possible measures of the risk. A linear combination of two *beta* was used in those cases in which the series *H* showed a bimodal character. The Kolmogorov-Smirnov test carried out on 20 daily time series proved the model fits the Hölder exponent very well in 70% of the cases. After simulating three high risk and four low risk portfolios we calculated and modeled their Hölder exponent. The results are remarkably interesting, through *H* and the *beta* distribution we are able to give a quantitative measure of the risk: shares and stock indexes have lower *H* mean values than EU bonds which proved to be low risk assets; the Arca Bonds Emergenti, made of non OECD bonds, had the highest mean value of all portfolios, however, it showed a much higher variance than all the others, which reflects the transitory turbulences emerging countries financial markets go through.

The developed model can be used to quantitatively evaluate the risk associated to pension funds portfolios. Further developments include using the parameters of Beta mixture to identify tolerable risk profiles and monitoring the investment portfolios of pension funds.

## Supporting Information

S1 Dataset S1 Dataset contains the 20 daily price series used in the experiments (See Table 1). (XLSX)

S2 Dataset S2 Dataset contains the series of the estimated H values for the 20 assets (Table 1) and for the seven portfolios (See Tables 2 and 3). (ZIP)

S1 Source Code S1 Source Code contains the source code to run the goodness of fit test for the mixture of Beta distributions. (PDF)
