# Supplementary material for: Financial Crisis: A New Measure for Risk of Pension Fund Portfolios
Source: PLoS One. 2015 Jun 18;10(6):e0129471. doi: 10.1371/journal.pone.0129471 (PMC4473272; doi:10.1371/journal.pone.0129471)
Supplement: S1 Source Code — (PDF) [file pone.0129471.s003.pdf]

```
##### Source code for the fitting of the linear combination of beta to the
##### empirical data and the goodness of fit test
##### Functions (0)-(4) written by Giamabattista Salinari, the rest by Marinella
##### Cadoni on December 2014.



##Functions for the fitting of a linear combination of beta.


# (0) Distribution function of a linear combiantion of beta

BetaMixture.p <- function(Q=0.5, a.1=5, b.1=1, a.2=2, b.2=2,  w=0.5){
  p.mixt <- w*(pbeta(q=Q,shape1=a.1, shape2=b.1)) + (1-w)*(pbeta(q=Q,shape1=a.2,
shape2=b.2))
  return(p.mixt)
}

# (1) Density function of a linear combination of beta

BetaMixture.d <- function(X=1.5, a.1=5, b.1=1, a.2=2, b.2=2,  w=0.5){
  if(w>=0 & w<=1){
    d.mixt <- w*(dbeta(x=X,shape1=a.1, shape2=b.1)) + (1-
w)*(dbeta(x=X,shape1=a.2, shape2=b.2))
  } else {
    d.mixt <- rep(0,length(X))
  }
  return(d.mixt)
}


# (2) Maximum likelyhood for a linear combination of beta

Beta2.ML <- function(Par=c(5, 1, 2, 2, 0.4), x=Beta2.dist){
  if(Par[5] > 0.5){
    ML <- -Inf
  } else {
    dens <- BetaMixture.d(X=Beta2.dist, a.1=Par[1], b.1=Par[2], a.2=Par[3],
b.2=Par[4], w=Par[5])
    ML <- sum(log(dens))
    return(ML)
  }
}




# (3) Fitting of a linear combination of beta

Beta2ParFinder <- function(Par){
  Pars <- optim(par=Par, Beta2.ML, control=list(fnscale=-1)) #, method="L-BFGS-
B", lower=c(0.1,0.1,0.1,0.1,0), upper=c(50,50,50,50,0.49999))
  return(Pars)
}




# (4) Selection of the initial parameters of the linear combination of beta

Beta2InitParFinder <- function(N=100, Min=0, Max=10){

  A.1 <- runif(N, Min, Max)
  B.1 <- runif(N, Min, Max)
  A.2 <- runif(N, Min, Max)
  B.2 <- runif(N, Min, Max)
```

```
  W <- runif(N, 0, 0.5)

  MLs <- vector()

  for(i in 1:N){
    Par.i <- c(A.1[i], B.1[i], A.2[i], B.2[i], W[i])
    MLs <- c(MLs,Beta2.ML(Par.i))
  }

  best <- which(MLs==max(MLs))[1]

  best.selection <- c(A.1[best], B.1[best], A.2[best], B.2[best], W[best])

  return(best.selection)

}



###### Test of goodness of fit of the linear combination of beta according to
###### the work of Clauset et al.,
###### see [17] in bibliography of our manuscript.


# INPUT: Beta2.dist : series of H values
# INPUT: B2par :  vector (a_1, b_1, a_2, b_2, w) with parameters indicated in
# table 1 of manuscript.

d=length(Beta2.dist)

#Apply ks-test to mixture with paramerts B2par obtained from data

t = ks.test(Beta2.dist, 'BetaMixture.p', B2par[1], B2par[2], B2par[3], B2par[4],
B2par[5])

count=0
limit=1500
random.ksstat=NULL

#Apply Monte Carlo procedure

for (i in 1:limit) {

    #generate random vector with values in [0,1]
    rx=runif(d,0,1)
    #evaluate original mixture of beta on the random vector to get a random
    #sample of mixture of beta
    Beta2.dist = rep(NA,d)

    for(j in 1:d){
        if(rx[j]<B2par[5]){
         Beta2.dist[j] = rbeta(1,B2par[1],B2par[2])
        }else{
        Beta2.dist[j] = rbeta(1,B2par[3],B2par[4])
         }
    }

    #generate new parameters of mixture of beta from the random sample

    Par = Beta2InitParFinder(N=10000, Min=1, Max=200)

    Rpar=Beta2ParFinder(Par)

    #apply ks test on the random sample and new mixture
```

```
    t2 = ks.test(Beta2.dist, 'BetaMixture.p', Rpar$par[1], Rpar$par[2],
Rpar$par[3], Rpar$par[4],Rpar$par[5]);

    random.ksstat <- c(random.ksstat,t2$stat)

    #count if new ks-test statistic is greater than original ks-test statistic
    if(t2$stat >= t$stat) {count = count + 1};

  }

# Visualise results

hist(random.ksstat,nclass=40)
abline(v=t$stat,lty=2,col=2)
mean(random.ksstat>=t$stat)

stat = t$statwarnings

#Original p-value
KSp = t$p

#p-value after Montecarlo simulation:
p = count/(limit)
```
